# Improved outcome in patients following autologous stem cell transplantation for multiple myeloma in south eastern Norway 2001–2010: a retrospective, population based analysis

**DOI:** 10.1186/s12885-018-4722-x

**Published:** 2018-08-08

**Authors:** Jon-Magnus Tangen, Geir Erland Tjønnfjord, Nina Gulbrandsen, Tobias Gedde-Dahl, Espen Stormorken, Kristina Anderson, Camilla Dao Vo, Fredrik Hellem Schjesvold, For Oslo Myeloma Center

**Affiliations:** 1 Department of Acute Medicine, Oslo University Hospital, P.O.Box 4950 Nydalen, 0424 Oslo, Norway; 2 Department of Haematology, Oslo University Hospital, P.O.Box 4950 Nydalen, 0424 Oslo, Norway; 3 Institute of Clinical Medicine, University of Oslo, P.O.Box 1171 Blindern, 0318 Oslo, Norway; 4 Department of Haematology, Akershus University Hospital, P.O.Box 1000, 1478 Lørenskog, Norway; 5 Department of Oncology, Section of Haematology, Østfold Hospital, P.O.Box 300, 1714 Grålund, Norway; 6 Department of Medicine, Innlandet Hospital, Gjøvik, P.O.Box 104, 2381 Brummundal, Norway

**Keywords:** Multiple myeloma, Autologous stem cell transplantation, Overall survival, Novel drugs

## Abstract

**Background:**

With the advent of novel drugs improved overall survival in patients with multiple myeloma, including patients who received up-front autologous stem cell transplantation (ASCT), has been reported from several centers. Here we report on overall survival in a population-based cohort of patients receiving ASCT as first line treatment and in whom novel agents were an option for second and later lines of treatment.

**Methods:**

Patients with multiple myeloma ≤ 65 years of age who were considered for ASCT from 01.01.2001–31.06.2005 (period 1) and from 01.07.2005 until 31.12.2009 (period 2) at Oslo University Hospital (OUH) were identified. Relevant data were collected from the patients’ medical records.

**Results:**

Altogether, 293/355 patients received ASCT. In all, median OS was 82.9 months in patients ≤ 60 years of age and 59.0 months in patients 61–65 years. For patients ≤ 60 years of age median OS increased from 70.6 months to 87.7 months (*p* = 0. 22) and median survival after start of second line therapy increased from 34.5 months to 46.5 months (*p* = 0.015) between the two periods. For patients 61–65 years of age median OS increased from 57.3 months to 61.2 months (*p* = 0. 87) and median survival after start of second line therapy was practically unchanged (32.6 months vs. 33.1 months (*p* = 0.97) between the periods. In patients ≤ 60 years of age salvage ASCT was used in 34% of the patients while in patients 61–65 years of age salvage ASCT was used in 7.3% of the patients. The use of salvage ASCT and novel drugs, as well as the number of treatment lines, were higher in patients ≤ 60 years of age and increased during the study period.

**Conclusion:**

In patients ≤ 60 years of age an increased median OS of 17 months between the two periods were noted, but the difference failed to reach statistical significance. However, a statistically significant difference in median survival of 12 months after start of second line therapy was found in this age group, which may be explained by a more active second line treatment. In patients 61–65 years only a slight increase of survival, not statistically significant, was noted between the periods.

## Background

Multiple myeloma is a neoplastic disorder caused by malignant transformation of plasma cells. The main clinical features are lytic bone lesions, bone marrow failure, and renal failure [1]. The incidence of multiple myeloma in Norway for the period 2001–2005 was 9.7/100000 for males and 6.5/100000 for females. Median age at diagnosis was 71.2 years for males and 69.3 years for females (Cancer Registry of Norway). The majority of patients respond to chemotherapy, but virtually all patients relapse and median survival with conventional chemotherapy was around three years [2]. In the 1990s autologous stem cell transplantation (ASCT) was introduced as first line treatment for patients below 65 years of age. The superiority of ASCT over conventional chemotherapy was first demonstrated in the French IFM study, which showed a median overall survival (OS) and a median event free survival of 57 and 44 months, respectively, for patients receiving ASCT, compared to 44 and 18 months, respectively, for patients receiving conventional chemotherapy [3]. These results were later confirmed by a British study [4]. Since the late 1990s ASCT has been standard first line treatment for myeloma patients < 65 years of age, based both on clinical efficacy [5] and effect on quality of life [6]. Between 1994 and 2000 the Nordic Myeloma Study Group conducted two population based studies on the clinical impact of ASCT, which in patients ≤ 60 years of age showed a median OS of 63 months in the ASCT group versus 39 months in a historical control group [7] and in patients 61–65 years of age showed a median OS of 50 months in the ASCT group versus 27 months in historical controls [8]. Since the late 1990s several new drugs have been introduced in the treatment of multiple myeloma, starting with the immunomodulatory drug (IMiD) thalidomide in 1999 [9], followed by the proteasome inhibitor bortezomib in 2003 [10] and the second generation IMiD, lenalidomide, in 2005 [11]. Late in the study period, new drugs such as the third generation IMiD pomalidomide [12], the second generation proteasome inhibitor carfilzomid [13] and the alkylator bendamustin [14] became available. In the context of ASCT the novel drugs are nowadays being used both as induction and consolidation treatment, as well as in first relapse or later lines of therapy. Their positive impact on survival in patients receiving ASCT has been shown in several multicenter clinical trials (reviewed in [15]). Also, several recent population-based studies have shown a steady improvement of survival in multiple myeloma in general, particularly in patients ≤ 60 years of age [16, 17].

On this background, we performed a population-based analysis of the treatment results in multiple myeloma in patients 65 years of age or younger in the South-East Health Region of Norway diagnosed in a nine-year period starting from 2001. According to the national Norwegian treatment guidelines ASCT was the preferred first line treatment for multiple myeloma in patients ≤ 65 years of age in this period. These patients were accordingly referred to the regional treatment centres for ASCT, with very few exceptions.

## Methods

A search was made in the patient administrative system at Oslo University Hospital (OUH) to identify patients ≤ 65 years of age who had been referred to the hospital with a diagnosis of multiple myeloma (C90.0 in the ICD 10 diagnostic system), and considered for ASCT in the period 01.01.2001–31.12.2009. The patient records of the selected patients were reviewed. OUH is the regional reference centre for the South-East Health Region of Norway (population: 2.9 million). Follow-up data were collected from patients’ records at OUH and referring hospitals.

### Treatment

#### Induction treatment

Induction treatment in the period was either vincristine, doxorubicin and dexamethasone (VAD) or cyclophosphamide and dexamethasone (Cy/Dex) [18], with the exception of two patients who received bortezomib/ dexamethasone.

#### Stem cell mobilizing therapy

Cyclophosphamide 2000 mg/m^2^ i.v. and G-CSF from day 4.

#### High dose therapy

Melphalan 200 mg/m^2^ (140 mg/m^2^ in case of creatinine clearance < 30 ml/min/m^2^). In five patients tandem transplantation was performed.

#### Consolidation

Between October 2005 and April 2009 eligible and consenting patients were randomized between no consolidation or consolidation with bortezomib [19]. Also, in the beginning of the study period consolidation with α-interferon was given. This treatment was part of the protocol in the previous Nordic ASCT studies [7, 8] but was later gradually abandoned in routine clinical practice, mainly because of it’s negative impact on quality of life. Furthermore, from 01.08.2009 until 31.11.2010 eligible and consenting patients were included in a clinical study and randomized to receive adjuvant treatment with the medicinal mushroom product Andosan™, which mainly contains the edible *Basidiomycetes* mushroom *Agaricus blazei* Murill, or with placebo [20].

*Patients not receiving ASCT* were treated at the discretion of the responsible physician (i.e. not by protocol).

*Second line treatment* was provided at the discretion of the responsible physician (i.e. not by protocol).

Patients were considered candidates for a second ASCT if the time from first ASCT to second line treatment was > 12 month.

### Diagnosis, response evaluation, disease progression

Diagnostics and response evaluation were based on the criteria applied by the Nordic Myeloma Study Group in previous ASCT studies [21], with some minor modifications:

*The diagnosis* of multiple myeloma was accepted if criteria A + C, A + D or B + C + D of the following was accepted: (A) serum monoclonal component (M-protein) concentration of immunoglobulin IgG > 30 g/l, IgA > 20 g/l, the presence of M-protein IgD or IgE regardless of concentration or Bence-Jones proteinuria > 1 g/l. (B) M-protein in serum or urine at lower concentration than described under A; (C) at least 10% plasma cells in bone marrow aspirate or biopsy verified plasmacytoma of bone or soft tissue; and (D) osteolytic bone lesions.

### Indication for ASCT

Only patients fulfilling criteria for treatment-demanding multiple myeloma were considered for ASCT or alternative chemotherapy.

### Treatment response

Complete response (CR) was defined as the disappearance of M-protein from serum and urine in agarose gel electrophoresis. Partial response (PR) was defined as at least 50% reduction of the initial serum M-protein concentration and a reduction of Bence-Jones protein to < 0.2 g/L. Minor response (MR) was defined as a 25% to 50% reduction of the initial serum M-protein concentration and a reduction of Bence-Jones protein by at least 50% but exceeding 0.2 g/L. The best response achieved at any time after ASCT was registered in the study. Progressive disease (PD) was defined as an increase of the M- component by ≥ 25%. Stable disease (SD) was defined as neither fulfilling any response criteria nor criteria for progressive disease.

### Classification

The patients were grouped according to the Durie and Salmon classification [22] and also according to the ISS classification [23] in cases where serum β2-microglobulin and serum albumin at diagnosis were available.

### Survival

*Total survival* was the time between the date of diagnosis and follow-up (01.05.2017) or death. For patients who were lost to follow-up total survival was the time from the date of diagnosis until last control.

*Time to next treatment* was the time from the date of diagnosis until start of second line treatment, or follow-up. This parameter is based on the clinical decision by the responsible physician to start treatment and not on the fulfillment of formal criteria for disease progression.

*Survival after start of second line treatment* was the time from start of second line treatment until follow-up or death.

### Statistics

Statistics was performed by the IBM SSPC 23 computer program. Survival analyses were performed by the Kaplan-Meier method. The median values and 95% confidence interval (CI) are indicated. Differences in survival were calculated by the Log Rank and Wilcoxon tests. Comparisons of the number of treatment regimens used in various time periods were done by the Independent samples t-test.

## Results

A search in the patient administration system of OUH identified a total of 623 patients ≤ 65 years of age with a diagnosis of multiple myeloma (C 90.0 in the ICD 10 diagnostic system) between 01.01.2001 and 31.12.2009. After review 268 patients were excluded (monoclonal gammopathy of uncertain significance, solitary myeloma, multiple myeloma with no treatment indication, primary plasma cell leukemia, treatment started before 01.01.2001, AL-amyloidosis, other types of hematologic malignancies, patients not residing in the South-East Health Region of Norway). A total of 355 treatment-demanding patients with multiple myeloma were included in the study, 293 patients received ASCT and 62 patients received other types of treatment. Three patients of foreign origin, who returned to their countries after ASCT, were censored for survival at the last control in Norway. No other patients were lost to follow-up. For patients offered ASCT, type of M-component and clinical stage at diagnosis are presented in Table 1 and treatment details and treatment response are presented in Table 2. The reasons for not giving ASCT were: comorbidity (31 patients), insufficient stem cell harvest (11 patients), complications to induction treatment (10 patients), disease progression (5 patients), earlier chemotherapy for other type of cancer (1 patient), no consent (4 patients).

**Table 1:** Patient chararcteristics-patients receiving ASCT

	Patients ≤ 60 years of age	Patients 61–65 years of age
	*N* (%)	*N* (%)
M-Component		
IgG κ	76 (36,1)	31 (37,5)
IgG λ	24 (11,9)	11 (13,8)
IgA κ	27 (12,0)	13 15,9)
IgA λ	12 (6,6)	7 (8,3)
light chain κ	42 (19,1)	10 (13,2)
light chain λ	16 (7,3)	5 (6,3)
non secretory	13 (6,0)	2 (2,5)
biclonal	2 (1,0)	0 (0)
no information	0	2 (2,5)
Stage Durie&Salmon		
IA	55 (26,4)	22 (27,1)
IB	5 (2,4)	2 (2,4)
IIA	82 (38,9)	33 (41,0)
IIB	16 (7,4)	4 (5,0)
IIIA	40 (18,5)	17 (21,2)
IIIB	14 (6,4)	3 (3,3)
Stage ISS		
ISS I	65 (31,0)	21 (26,3)
ISS II	40 (18,5)	18 (22,6)
ISS III	39 (18,1)	19 (23,8)
No information^*)^	68 (32,4)	23 (27,3)

*)β-globulin missing

**Table 2:** Treatment characteristics- Patients receiving SCT

	Patients ≥ 60 years of age	Patients 61–65 years of age
	*N* (%)	*N* (%)
Induction treatment		
VAD	90 (42,6)	32 (39,5)
Cy/Dex	120 (56,5)	49 (60,5)
Vel/Dex	2 (0,9)	0
Consolidation		
IFN	38 (17,9)	10 (12,3)
Bortezomib	25 (11,9)	7 (8,6)
No consolidation	149 (70,2)	64 (79,1)
Treatment response		
Progressive disease	1 (0,6)	3 (3,6)
Stable disease	7 (3,2)	1 (1,2)
Minimal response	9 (4,2)	2 (2,4)
Partial response	102 (48,1)	49 (59.0)
Complete response	57 (27,2)	19 (22,9)
Not evaluable	36 (16,7)	9 (10,9)

*VAD* vincristin- adriamycin-dexamethasone, *Cy/Dex* cyclophosphamide-dexamethasone, *Vel /Dex* Bortezomib-dexamethasone, *IFN* α- Interferon

### Survival

#### Overall survival patients ≤ 60 years of age

**Fig. 1:**
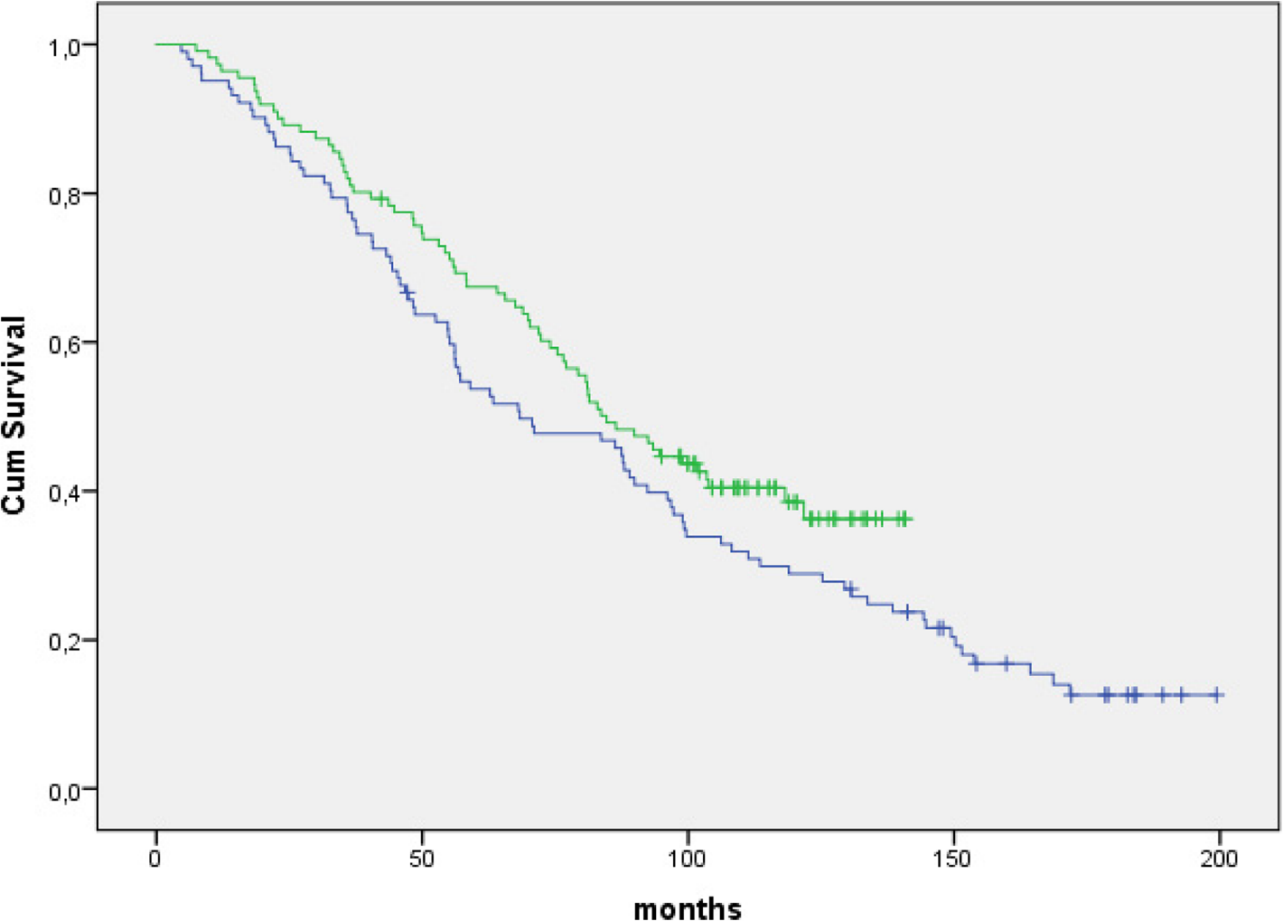
Overall survival in patients ≤ 60 years of age receiving ASCT. Blue curve: Patients who started treatment in the period 01.01.2001–31.06.2005 (*n* = 99). Median overall survival: 70.6 months (95% CI 53.2–88.1). Green curve: Patients who started treatment in the period 01.07.2005–31.12.2009 (*n* = 113). Median overall survival: 87.7 months (95% CI 75.2–100.1). *P* = 0.21 (ns)

In the study period 233 patients started treatment for multiple myeloma, 212 (91%) patients received ASCT and 21 (9%) patients received other treatments. Two patients refused ASCT and 19 patients did not receive ASCT because of comorbidity or for other clinical reasons. Median OS for all patients was 75.4 months (95% CI 63.5–87.3), 82.9 months (95% CI 70.8–95.0) for patients receiving ASCT and 27.0 months (95% CI 17.0–37.1) for patients not receiving ASCT (*p* < 0.0001). For patients receiving ASCT and starting therapy between 01.01.2001 and 31.06.2005 (*n* = 99) median OS was 70.6 months (95% CI 53.2–88.1), while median OS was 87.7 months (95%CI 75.2–100.1) for patients receiving ASCT and starting therapy between 01.07.2005 and 31.12.2010 (*n* = 113). Thus, the median overall survival increased by 17 months between these two periods (Fig. 1). However, this difference was not statistically significant (*p* = 0.22).

#### Patients 61–65 years of age

In the study period 122 patients in this age group started treatment, 81 (66%) patients received ASCT and 41 (34%) patients received other treatments. Two patients refused ASCT and 39 patients did not receive ASCT because of comorbidity or for other clinical reasons.

**Fig. 2:**
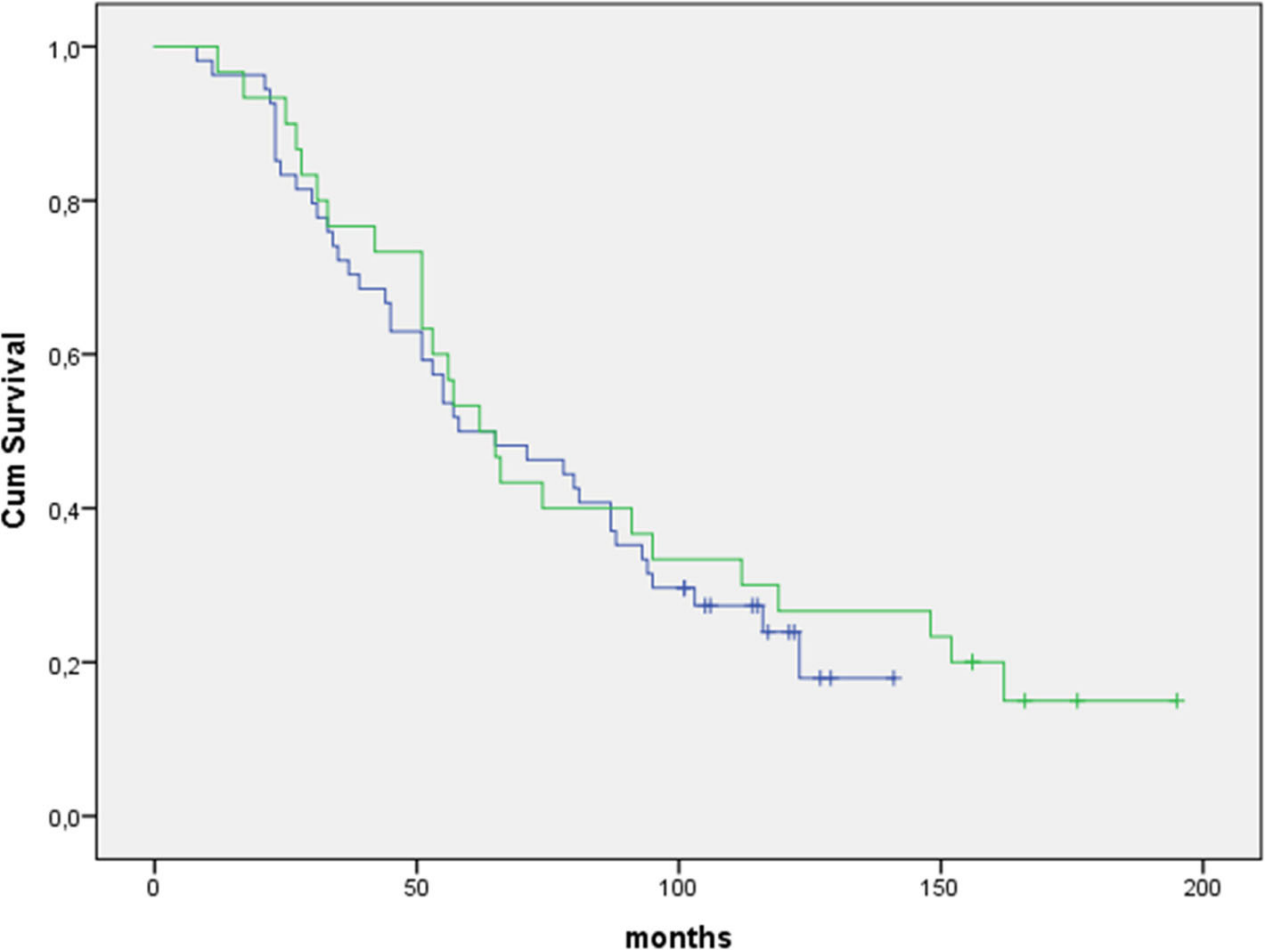
Overall survival in patients 61–65 years of age receiving ASCT. Blue curve: Patients who started treatment in the period 01.01.2001–31.06.2005 (*n* = 32). Median overall survival = 57.3 months. Green curve = Patients who started treatment in the period 01.07.2005–31.12.2009 (*n* = 49). Median overall survival = 61.2 months. (*p* = 0,87(ns))

Medan OS for all patients in this age group was 46.0 months (95% CI 37.5–54.5). For patients receiving ASCT median OS was 59.0 months (95% CI 43.0–76.6) and for the other patients median OS was 31.6 months (95% CI 15.1–48.1) (*p* < 0.0001). For the patients starting treatment in the period 01.01.2001–31.06.2005 and receiving ASCT, median OS was 57.3 months (95% CI 45.2–69.4) (*n* = 32), while median OS was 61.2 months (95% CI 29.7–92.7) for patients receiving ASCT and starting treatment in the period 01.01.2005–31.12.2009 (*n* = 49). Thus, only a small improvement of overall survival with approximately 4 months between these two periods for patients receiving ASCT was noted (*p* = 0.87) (Fig. 2).

### Second line therapy

#### Patients ≤ 60 years of age

Second line therapy was performed in 187/212 patients ≤ 60 years of age who received ASCT as first line therapy (88%). Mean age at start of second line therapy was 57.3 years. Of the 25 patients who did not receive second line therapy two patients died within 90 days of ASCT (early death) and six patients died from non-myeloma disease. The remaining 17 patients were alive without disease progression at follow-up. In total, 64 patients (34.2%) received salvage ASCT. Their median age was 56.5 years. Median time to second line therapy was 36.1 months (95% CI 33.3–38.8) in patients who started treatment between 01.01.2001 and 31.06.2005 (*n* = 89) and 33.5 months (95% CI 27.3–39.4) in patients who started treatment between 01.07.2005 and 31.12.2009 (*n* = 98). Thus, the time to new treatment practically did not change during the study period. For patients starting treatment in the first period median survival after start of second line therapy was 34.5 months (95% CI 23.6–45.3), while for patients who started treatment in the second period median survival after start of second line therapy was 46.5 months (95% CI 36.8–56.2) (*p* = 0.015).

**Fig. 3:**
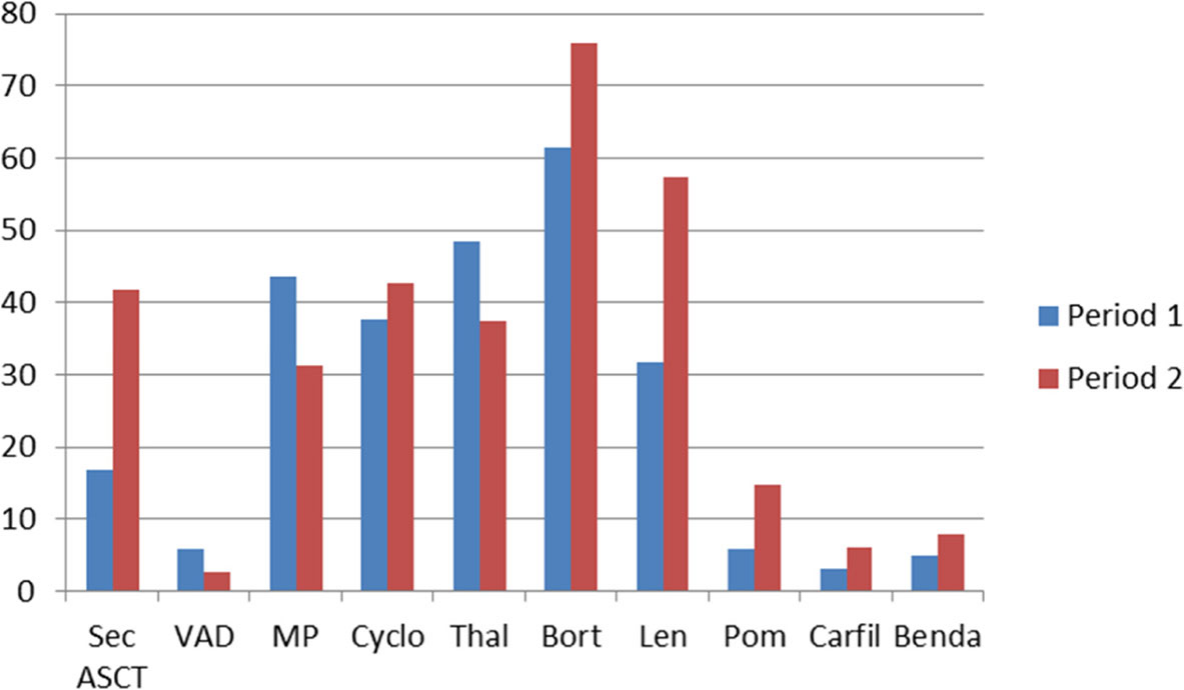
Second and later lines of treatment. Patients ≤ 60 years of age. Percentage of patients given different treatments in the two periods. Period 1 = 01.01.2001–31.06.2005 Period 2 = 01.07.2005–31.12.2009. Sec ASCT = Salvage ASCT, VAD = Vincristin-Adriamycine-Dexamethasone, MP = Melphalan –Prednisolone, Cyclo = Cyclophosphamide, Thal = Thalidomide, Bort = Bortezomib, Len = Lenalidomide, Pom = Pomalidomide, Carfi = Carfilzomide, Benda = Bendamustine

Figure 3 shows the percentage of patients given different second line treatment regimens in the two periods. In the second period there was a significant increase of the use of salvage ASCT, from 18.8% (17/90 patients) to 41.7% (47/97 patients) of the patients) (*p* < 0.0001), an increase in the use of bortezomib (from 61.4 to 76.0%) (*p* = 0.1), lenalidomide (from 31.7 to 57.4%) (*p* < 0.001) and pomalidomide (from 5.7 to 14.8%) (*p* = 0.02), as well as a decreased used of melphalan from 48,1% to 31,9% (*p* = 0,01) and thalidomide from 48,0% to 36,3% (*p* = 0,1), compared to the first period.

The median number of lines of therapy given beyond first line was 2.96 in the first period and 3.63 in the second period (*p* = 0.002).

#### Patients 61–65 years of age

Among the 81 patients who received ASCT, second line therapy was started in 65 patients (80%). Mean age at start of second line therapy was 65.5 years. Of the 16 patients who did not receive second line treatment two patients died within 90 days from ASCT (early death) and six patients died from non-myeloma related causes. The remaining eight patients were alive without disease progression at follow-up. Six patients received salvage ASCT (9.2%). Median time to second line therapy was 35.7 months (95% CI 24.0–47.9) in patients who started treatment between 01.01.2001 and 31.06.2005 (*n* = 32) and 37.3 months (95% CI 20.7–53.9) in patients who started treatment between 01.07.2005 and 31.12.2009 (*n* = 49) (*p* = 0,82). Median survival after start of second line therapy was 32.6 months (95% CI 21.7–42.9)(*n* = 31) in the first group and 33.1 months (95% CI 19.3–46.9) in the second group (*n* = 49) (*p* = 0,97).

**Fig. 4:**
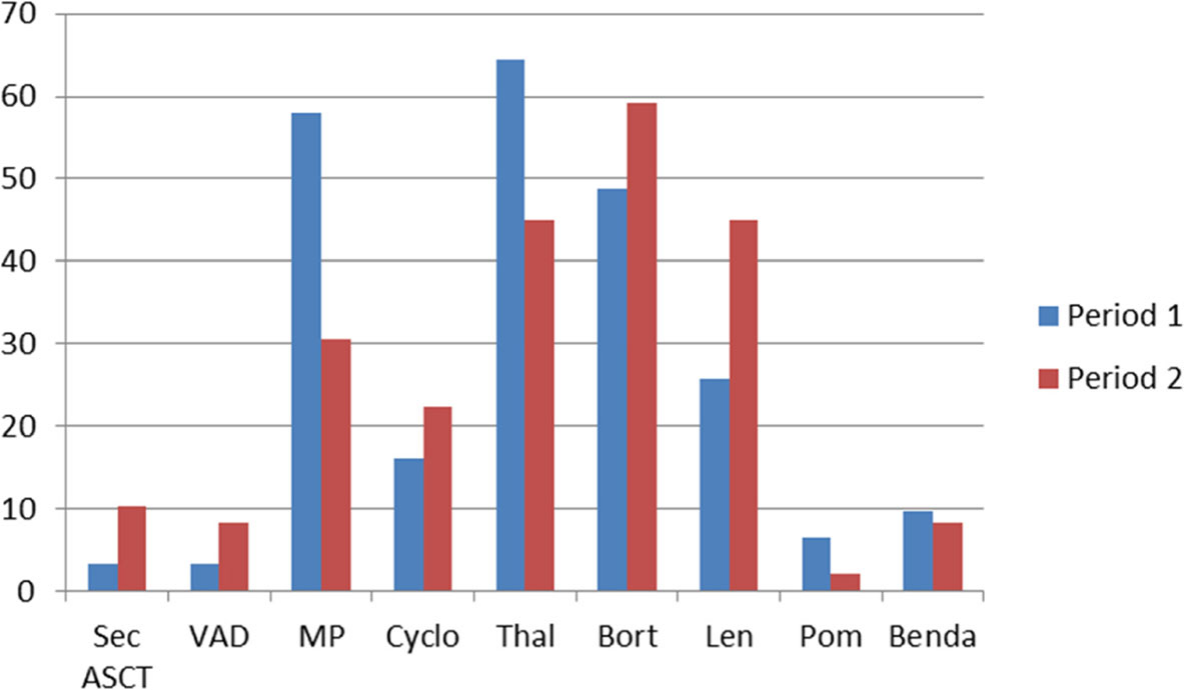
Second and later lines of treatment. Patients 61–65 years of age. Percentage of patients given different treatments in the two periods. Period 1 = 01.01.2001–31.06.2005 Period 2 = 01.01.2005–31.12.2009. Sec ASCT = Salvage ASCT, VAD = Vincristin-Adriamycine- dexamethasone, MP = Melphalan- Prednisolone, Cyclo = Cyclophosphamide, Thal = Thalidomide, Pom = Pomalidomide, Benda = Bendamustin

Figure 4 shows the percentage of patients given different second line treatment regimens in the two periods in this age group. Salvage ASCT was used in 9.2% (6/65) of the patients, and there was an increased use of salvage ASCT from 3.7 to 13.1% between the study periods (1/27 patients vs. 5/38 patients). Furthermore, a decrease in the use of melphalan/prednisolone (from 64.5 to 30.6%) (*p* = 0,01) and thalidomide (from 64.5 to 44.9%) (*p* = 0,09) as well as an increased use of bortezomib (from 48.4 to 59.2%) (*p* = 0,15) and lenalidomide (from 25.8 to 44.9%) (*p* = 0,02), was noted between the study periods.

The median number of lines of therapy delivered beyond first line was 2.84 in the first period and 3.00 in the second period (*p* = 0,71).

### Influence of CR versus non CR after ASCT on survival parameters

In patients < 60 years CR was reached in 57/176 (27.2%) of the patients evaluable for response, while response was not evaluable in 36 patients. Statistically significant increased OS and time to second line therapy was found in CR patients compared to non CR patients in both treatment periods (Table 3). In patients 61–65 years CR was reached in 19/72 of the patients evaluable for response, while response was not evaluable in 9 patients. Also in this age group a trend for a survival advantage in CR patients was found (Table 4). However, the results must be interpreted with caution because of the low number of patients in this age group.

**Table 3:** Survival parameters - CR versus non CR after ASCT. Patients ≤ 60 years. Number of patients evaluable for treatment response = 176

	Median Overall Survival (Months) (95% CI)			Median Time to New Treatment (Months) (95% CI)		
	CR^1^	Non CR^2^	*P*- value	CR	Non CR	*P*-value
Period 1^3^ (*N* = 69)	92.3 (33.1–151.6)	56.1 (39.1–73.1)	*P* = 0.04	45.4 (7.0–110.3)	36.6 (31.3–42.0)	*P* = 0.001
Period 2^4^ (*N* = 107)	Not reached^a^	81.0 (76.1–113.3)	*P* = 0.001	49.7 (8.4–91.0)	29.4 (25.6–33.2)	*P* = 0.001
Period 1 + 2 (*N* = 176)	133.7 (80.6–186.9)	79.3 (62.2–96.3)	*P* = 0.001	49.7 (19.2–80.1)	31.3 (25.2–37.3)	*P* = 0.001

^a^Mean overall survival = 112.3 months (95% CI 99.7–124.4)

^1^Number of CR patients: Period 1: 24/69 Period 2: 33/107

^2^Number of non CR patients Period 1: 45/69 Period 2: 74/107

^3^Period 1 = 01.01.2001–31.06.2005

^4^Period 2 = 01.07.2005–31.12.2009

**Table 4:** Survival parameters - CR versus non CR after ASCT. Patients 61–65 years. Number of patients evaluable for treatment response = 72

	Median Overall Survival (Months) (95%CI)			Median Time to New Treatment (Months) (95%CI)		
	CR^1^	Non CR^2^	*P*- value	CR	Non CR	*P*-value
Period 1^3^ (*N* = 32)	59.8 (41.0–78.5)	52.1 (8.4–95.8)	*P* = 0.8 (ns)	28.7 (5.0.-51.4)	35.7 (14.9–56.5)	*P* = 0.8 (ns)
Period 2^4^ (*N* = 40)	75.0 (46.3–102.6)	50.1 (42.1–68.1)	*P* = 0.2 (ns)	56.4 (32.4–80.9)	29.9 (16.4–39.3)	*P* = 0.02
Period 1 + 2 (*N* = 72)	68.5 (51.7–85.4)	52.3 (48.1–56.1)	*P* = 0.4	43.9 (28.7–59.0)	31.3 (24.3–37.8)	*P* = 0.04

^1^Number of CR patients: Period 1: 6/32 Period 2: 13/40 Total 19

^2^Number of non CR patients Period 1: 26/32 Period 2: 27/40 Total 53

^3^Period 1 = 01.01.2001–31.06.2005

^4^Period 2 = 01.07.2005–31.12.2009

## Discussion

In this population based retrospective study of the outcome of ASCT in clinical practice a median OS of 82.9 months was found in patients ≤ 60 years of age and 59.0 months in patients 61–65 years of age. This represents an improved outcome compared to previous Nordic studies, conducted in the periods 1994–1997 and 1998–2000, respectively, which showed a median overall survival of 63 months in patients ≤ 60 years of age [7] and 50 months for patients 61–65 years of age [8]. Furthermore, our findings in this population-based study indicate an improved survival during the study period as median OS increased from 70.6 months to 87.7 months for patients ≤ 60 years of age. However, the difference failed to reach statistical significance, which may be explained by the heterogenicity of this population based patient material, which results in larger confidence intervals than usually encountered in prospective clinical studies with strict inclusion criteria. In patients 61–65 years of age, median OS only increased from 57.3 months to 61.2 months between the periods. An inferior survival in the age group 61–65 years of age compared to patients ≤ 60 years of age was previously reported in a Nordic study [8], and recently a similar result was found in a comprehensive analysis of 2316 patients 61–65 years of age included in studies conducted by the Intergroup Francais de Myelome [24]. Time to new treatment remained approximately the same in both age groups and in both study periods. This indicates that the net difference noted in overall survival was due to differences in the results of salvage therapy following relapse. In the younger age group median OS after start of salvage therapy increased from 33,5 months to 46,5 months between the two periods (*p* = 0.015), whereas OS practically did not change in the higher age group (32.6 months vs 33.1 months). In patients ≤ 60 years of age 34.3% of the patients received salvage ASCT and the use of salvage ASCT increased significantly between the two periods, from 16.8 to 41.7%. In the higher age group the use of salvage ASCT increased only from 3.7 to 13.1% between the two periods. The differences in the use of salvage ASCT may be explained by the differences in age at start of second line therapy (mean age was 57.3 years in patients ≤ 60 years of age and 65.5 years in patients 61–65 years of age). In both groups an increased use of bortezomib, lenalidomide and pomalidomide was noted in the second period. However, the use of novel drugs was generally higher in patients ≤ 60 years of age than in the higher age group. Furthermore, in younger patients the number of treatment lines after progression increased between the two periods. This may be interpreted as a more active approach to treatment after progression in younger patients, both at OUH and at the other hospitals in the region, where a majority of the patients were followed after their first ASCT. Furthermore, our study shows a clear survival advantage of patients reaching CR after ASCT, versus non CR patients. This result is in line with previous reports from other population based studies [25, 26].

## Conclusion

The results in this study confirms other population based reports of increased survival in recent years in patients receiving ASCT [25, 27] and shows that patients with multiple myeloma in Norway benefit from improved treatment in routine clinical practice. The improvement is most pronounced in patients ≤ 60 years of age, which may be explained by an increased use of salvage ASCT and novel drugs; in other words a more active approach to treatment at progression in this age group.

## Abbreviations

ASCT: High dose chemotherapy with autologous stem cell support
CR: Complete response
IMiD: Immunomodulatory drug
MR: Minor response
OS: Overall survival
OUH: Oslo University Hospital
PD: Progressive disease
PR: Partial response
SD: Stable disease
